# Analysis of spatial and temporal dynamics of xylem refilling in *Acer rubrum* L. using magnetic resonance imaging

**DOI:** 10.3389/fpls.2013.00265

**Published:** 2013-07-22

**Authors:** Maciej A. Zwieniecki, Peter J. Melcher, Eric T. Ahrens

**Affiliations:** ^1^Department of Plant Sciences, University of California at DavisDavis, CA, USA; ^2^Biology Department, Ithaca CollegeIthaca, NY, USA; ^3^Department of Biological Sciences, Carnegie Mellon UniversityPittsburgh, PA, USA

**Keywords:** embolism, xylem, MRI imaging, refilling, tension

## Abstract

We report results of an analysis of embolism formation and subsequent refilling observed in stems of *Acer rubrum* L. using magnetic resonance imaging (MRI). MRI is one of the very few techniques that can provide direct non-destructive observations of the water content within opaque biological materials at a micrometer resolution. Thus, it has been used to determine temporal dynamics and water distributions within xylem tissue. In this study, we found good agreement between MRI measures of pixel brightness to assess xylem liquid water content and the percent loss in hydraulic conductivity (PLC) in response to water stress (P_50_ values of 2.51 and 2.70 for MRI and PLC, respectively). These data provide strong support that pixel brightness is well correlated to PLC and can be used as a proxy of PLC even when single vessels cannot be resolved on the image. Pressure induced embolism in moderately stressed plants resulted in initial drop of pixel brightness. This drop was followed by brightness gain over 100 min following pressure application suggesting that plants can restore water content in stem after induced embolism. This recovery was limited only to current-year wood ring; older wood did not show signs of recovery within the length of experiment (16 h). *In vivo *MRI observations of the xylem of moderately stressed (~-0.5 MPa) *A. rubrum *stems revealed evidence of a spontaneous embolism formation followed by rapid refilling (~30 min). Spontaneous (not induced) embolism formation was observed only once, despite over 60 h of continuous MRI observations made on several plants. Thus this observation provide evidence for the presence of naturally occurring embolism-refilling cycle in *A. rubrum*, but it is impossible to infer any conclusions in relation to its frequency in nature.

## INTRODUCTION

There is widespread agreement that negative hydrostatic pressures make water transport in the xylem intrinsically vulnerable to cavitation (Pickard, 1981; Tyree and Zimmermann, 2002). In order to maintain hydraulic capacity, plants must either minimize cavitation or restore conductivity in embolized conduits. The idea that embolized vessels might be returned to their functional state is not new, but it has generally been thought to be limited to situations in which the entire vascular system could be pressurized due to active solute transport by the roots (Fisher et al., 1997). However, more recent studies indicated that embolism removal may be possible even when the majority of the water in the xylem remains under low, or moderate tensions (Salleo et al., 1996; Canny, 1997; McCully et al., 1998; Zwieniecki and Holbrook, 1998; McCully, 1999). This triggered a substantial effort to provide a conceptual framework and descriptions of important prerequisites that could explain how xylem refilling could occur in actively transpiring plants (Holbrook et al., 1999; Tyree et al., 1999; Salleo et al., 2009; Zwieniecki and Holbrook, 2009; Nardini et al., 2011; Secchi and Zwieniecki, 2011).

Our current understanding of the spatial and temporal patterns of embolism formation and refilling relies heavily on measurements from destructive sampling techniques, such as measuring changes in stem hydraulic conductivity. However, there are several less invasive methods such as the use of a cryo-scanning electron microscope (cryo-SEM) that allows one to view the liquid (ice) content within the xylem of stems that were rapidly frozen in liquid nitrogen (Canny, 1997, 2001; McCully et al., 1998, 2000; Pate and Canny, 1999; Melcher et al., 2001). This cryo-SEM technique has helped to resolve some questions regarding the spatial distributions of embolism formation (Canny, 1997, 2001; McCully et al., 1998, 2000; Pate and Canny, 1999; Melcher et al., 2001). For example, they show that vessels tend to embolize in clusters, and that many embolized vessels had droplets of frozen water on their vessel walls. However, results from cryo-SEM studies were called into question because potential artifacts may arise during the freezing procedure (Cochard et al., 2000). A more recent study used high-resolution computed tomography to view *in vivo* water content in the stems on *Vitis vinifera* L. plants (Brodersen et al., 2010). Collected images showed not only the presence of water droplets on the walls of embolized vessels but also the dynamic changes in droplet size during refilling. These data provide strong support for the presence of refilling.

Studies that have investigated the temporal dynamics of the refilling process using artificially induced embolism show that refilling was more or less completed within an hour after embolism induction (Salleo et al., 1996; Zwieniecki et al., 2004; Secchi and Zwieniecki, 2011). Similar findings come from observations of natural embolism in petioles of red maple (*Acer rubrum* L.) and tulip trees (*Liriodendron tulipifera* L.) using a double staining method (Zwieniecki et al., 2000). However, reversal of embolism in vines (*Vitis *spp.) was observed to only occur when transpiration had been stopped (Zwieniecki et al., 2000; Holbrook et al., 2001). In addition, the temporal pattern of recovery from embolism seems to be related to the level of plant water stress. For example, *Laurus nobilis *L. and *A. negundo *L. only refilled embolisms over prolonged recovery times of 24 h and only when water stress levels were significantly reduced (Hacke and Sperry, 2003). Secchi and Zwieniecki (2011) showed that the rate of embolism recovery in poplar trees (*Populus trichocarpa* L.) was dependent on the level of water stress. Their study showed faster recovery (less than 2 h) in moderately stressed trees compared to much longer recovery times (more than 20 h) in trees exposed to severe stress. The difference in recovery rates was observed despite the fact that stem water potentials increased in both cases within 1 h (Secchi and Zwieniecki, 2011).

Most of the evidence that demonstrates rapid refilling in plants relies on destructive sampling methods that could be prone to methodological problems. The few *in vivo* observations using magnetic resonance imaging (MRI) and x-ray tomography show only very slow recovery in species with large vessels: *Vitis *spp.**(Holbrook et al., 2001; Brodersen et al., 2010) and *Cucumis sativus *(Scheenen et al., 2007). Thus, there is still a lack of *in vivo *evidence that would provide supporting evidence of the rapid rates of the embolism-refilling cycles observed in species with small vessels obtained using destructive sampling techniques. The goal of this short contribution is aimed specifically at addressing this issue. We present results of direct observations of naturally occurring embolism/refilling cycle in stems of *A. rubrum* observed using MRI.

## MATERIALS AND METHODS

Study was conducted on *A. rubrum* plants either 2-year-old plants with minimum 1 m long stem and branches collected from 20-year-old *A. rubrum* trees. For all of the MRI experiments, prior to placing a plant or a sample into the MRI magnet, a 15-mm diameter surface coil radio frequency resonator was placed on the stem. Each plant was positioned in an 11.7 T, 89 mm vertical-bore, Bruker AVANCE micro-imaging system. The sample temperature was regulated at ~25°C by pumping air through the magnet bore. For image data collection, we used a T2/spin-density-weighted 3D Fourier transform spin-echo sequence (T2W-3DFT) with a repetition time/echo time (TR/TE) = 980/45 ms. The T2W-3DFT data provided good free water versus air contrast. Images were acquired with a 256 × 128 × 128 matrix and then zero-filled to 512 × 256 × 256 before Fourier transformation, yielding a final isotropic resolution of approximately 50 μm. The imaging time was approximately 90 s per image with 90 s resting time between images.

To compare MRI analysis of xylem water content to stem hydraulic conductivity, we used ~2-m-long leafy branches that were collected from seven trees (15–20 years old) growing in the field at Harvard Forest. Several leaves on each branch were placed into sealed plastic bags and covered in aluminum foil the evening before collecting branches at predawn the next day. After excising branches in the air, they were allowed to continue to transpire (in the shade) until the loss of water from the uncovered leaves reduced covered leaf water potentials to values that were needed to generate a vulnerability to embolism response curve. Covered, branch equilibrated leaf water potentials were measured using a pressure chamber system. The balancing pressure required to squeeze water to the excised petiole surface was determined and used to estimate stem water potentials. Following dehydration, each branch was labeled and was double bagged in large black plastic bags. Wet paper towels lined the two-bag layers to reduce evaporation and to allow the branch water potentials to equilibrate within each sample. These branches were then shipped from Harvard Forest, Petersham, MA to the MRI facility in Carnegie Mellon University in Pittsburgh, PA.

Prior to MRI measurements, leaf water potentials were re-measured using the same pressure chamber system to determine equilibrated water potentials of the branch samples. For each sample, a long portion of the stem was excised under water first and then two 5-cm-long stem segments were subsequently excised underwater from the current extension growth (number of sample tested 25). One of the excised stem samples was used to determine the PLC using classical hydraulic pressure-flow methods. The other excised sample was used for the determination of the water content using MRI. The MRI sample was tightly wrapped in parafilm to further reduce desiccation during the measurement. After MRI imaging was complete, a post-processing image registration algorithm was applied to the data to correct for physical translations of the stem in the image field of view over the measurement time (total successful measurements 20). Image brightness was adjusted for all images using two control glass tubes filled with DI H_2_O and 1:1 mixture of DI H_2_O and D_2_O (volumetric). Pixel brightness ranged in images from black (0 value) to white (65525 value), and these values corresponded to increasing concentration of unbound water that was present in the voxel (volumetric picture element 50 μm × 50 μm × 1000 μm) and were used for analysis of xylem water content (Matlab12, MathWorks, Inc., Natick, MA, USA).

To determine the potential for spontaneous embolism formation in moderately stressed stems, undisturbed 3-year-old plants were fitted through the magnet bore using the same strategy as described above. Each plant was left in the magnet for 10–15 h and images were taken every 3min (90 s signal collection time). Images were acquired using a multi-slice gradient-echo sequence with TR/TE = 75/5 ms and a 512 × 256 × 256 matrix size, in-plane resolution of 50 μm × 50 μm and 1 mm thick slices. Images were simultaneously collected from five slices separated by 2 mm distance and thus covering a total of 15 mm of stem length. Data were analyzed using Matlab 12 (MathWorks). During the 10- to 15-h observation period, plants were not subjected to any experimental treatments or any disturbance. They were maintained at an average leaf water potential of about -0.5 MPa. Total time of observation equaled 60 h.

Long-term MRI observations were followed by an air-injection experiment to determine the temporal dynamics of artificially induced embolism in intact plants. Prior to attaching a pressure collar near the base of the main stem of each plant a small incision was made to allow pressurized gas to penetrate the xylem of the plants during air-injection (Crombie et al., 1985). Each of three plants was pressurized so that the pressure gradient across the bordered pit membranes equaled 5.0 MPa (sum covered leaf water potential and injection pressure). The pressure was held for 2 min. while the plant was still in the MRI magnet. MRI measurements were made during and after air-injection to determine if *A. rubrum* could recover from artificially induced embolism.

## RESULTS

**FIGURE 1:**
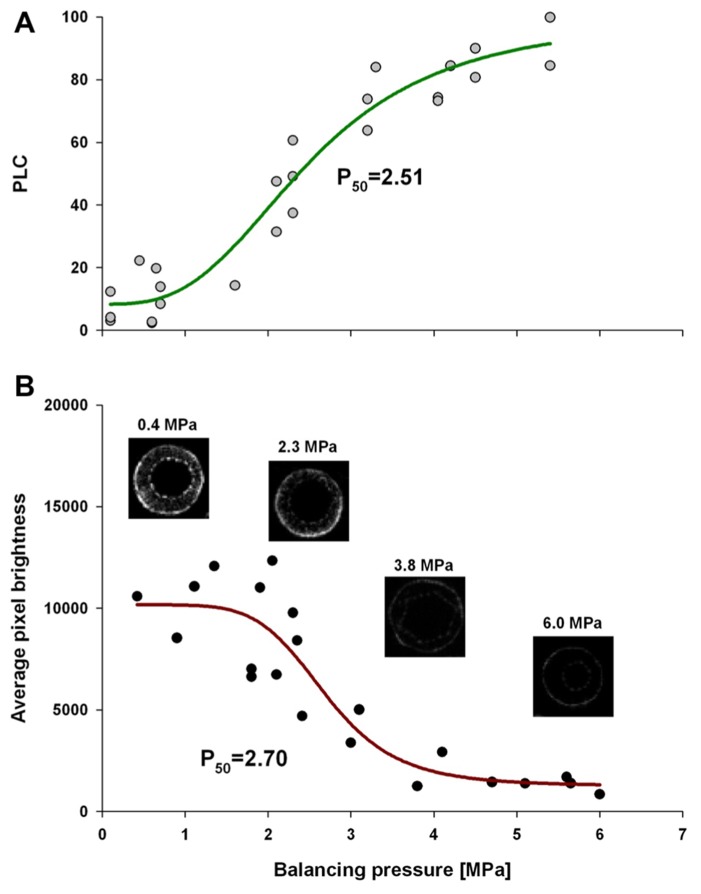
** Leaf water potential, measured on equilibrated branches, are plotted to PLC (A), and average pixel brightness (black = 0 to white = 65525) determined from MRI analysis (B), is shown.** Both data sets were fitted with a dose–response curve (solid line) in the form of PLC = min_PLC_ + (max_PLC_ - min_PLC_)/[1 + (C/EC_50_)^slope^], where min_PLC_ is minimum PLC in non-stressed plants, max_PLC_ is 100%, EC_50_ represents 50% loss of initial functionality [min_PLC_ + (max_PLC_ - min_PLC_)/2], and slope is the rateof PLC increase at EC_50_. There was no statistical difference between EC_50_ from two methods (PLC and MRI). The same function was used for pixel brightness curve fitting except that min_PLC_ and max_PLC_ were substituted with average pixel brightness at low and high ends of stem water stress. The four MRI images shown are representative images that were used to create the MRI-vulnerability curve. Only pixel brightness data from the xylem conducting area was used to produce the curve. All measurements were made on current-year extension growth.

Comparative analysis of xylem water content from MRI images and stem hydraulic measurements were made on current-year extension growth. Analysis was made on branches exposed to varying levels of water stress to assess the relationship of pixel brightness measured with MRI to changes in stem hydraulics. Pixel brightness measured with MRI is related to the amount of free water in the sample. In plant tissues, this would be the water that can freely move and is not bound within cellular walls. The generated MRI-based “vulnerability” curve was found to be similar to the PLC curve measured using hydraulic methods (**Figure 1**). We found that stress of -2.51 MPa was required to reduce the xylem hydraulic conductance of the xylem of current-year extension growth of *A. rubrum* plants by 50% (P_50_), determined from hydraulic methods. The equivalent 50% loss of average pixel brightness in MRI images was determined to be -2.70 MPa. We also observed similarities in the shape of the vulnerability to embolism curves obtained from both hydraulic methods and MRI image analysis and no statistical difference between estimates of EC_50_ (**Table 1**). These results provided assurance that pixel brightness was a good proxy for analysis of stem water content and for estimating changes in stem hydraulic conductance due to embolism formation (**Figure 1**).

**Table 1:** Statistical analysis of the fit of dose–response curve (**Figure 1**) (A) PLC = min_PLC_ + (max_PLC_ - min_PLC_)/[1 + (C/EC_50_)^slope^)] to PLC and (B) pixel brightness (pb) from MRI {pb = min_pb_ + (max_pb_ - min_pb_)/[1 + (C/EC_50_)^slope^]}.

A. PLC method
*R* = 0.96802847	*R*^2^ = 0.93707912	Adjusted *R*^2^ = 0.92849900
**Parameter estimates**
	**Coefficient**	**SE**	***t***	***P***
min	8.3246	3.3956	2.4516	0.0226
max	100.0000	13.9177	7.1851	<0.0001
EC50	2.5167	0.3125	8.0525	<0.0001
Hillslope	-2.9890	1.0380	-2.8795	0.0087
**Analysis of variance:**
	**DF**	**SS**	**MS**	***F***	***P***
Regression	3	25308.9213	8436.3071	109.2152	<0.0001
Residual	22	1699.3865	77.2448
Total	25	27008.3078	1080.3323
**B. MRI - Pixel brightness**
*R* = 0.90873197	*R*^2^ = 0.82579380	Adjusted *R*^2^ = 0.79505153
**Parameter estimates**
	**Coefficient**	**SE**	***t***	***P***
Minimum	1262.0805	945.4022	1.3350	0.1995
Maximum	10171.8589	908.9276	11.1911	<0.0001
EC50	2.7009	0.2490	10.8459	<0.0001
Hillslope	6.2416	2.8575	2.1843	0.0432
**Analysis of variance:**
	**DF**	**SS**	**MS**	***F***	***P***
Regression	3	270109225.7591	90036408.5864	26.8618	<0.0001
Residual	17	56981175.6885	3351833.8640
Total	20	327090401.4476	16354520.0724

**FIGURE 2:**
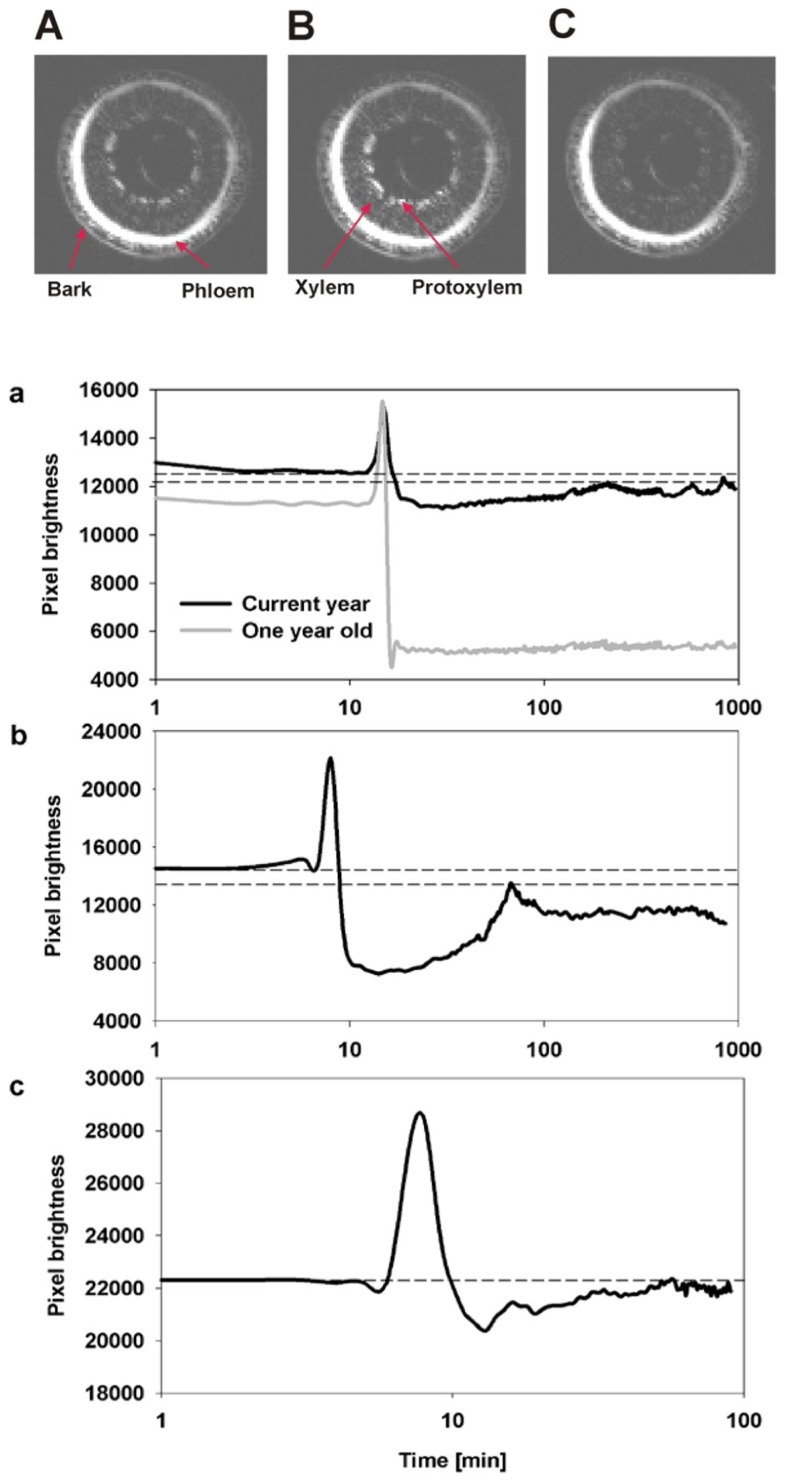
** Representative MRI images of three consecutive observations of the state of water status of xylem from current-year extension growth (A) before air injection, (B) during air injection, and (C) 10 min after air injection are shown.** The images **(A,C)** reflect the changes in pixel brightness after the successful induction of embolism from air injection. The temporal increase of brightness observed in image **(B)** compared to **(A,C)** reflects the movement of water during the injection of air into the stem. The three figures below **(a–c)** are an analysis of changes in the average pixel brightness measured on the xylem conducting area from three experimental plants. Panel **(a)** displays analysis of two xylem growth rings simultaneously, and shows a lack of refilling in the 1-year-old ring as seen by the drop in average brightness to a new lower constant level while refilling was observed in the current-year growth ring as shown by the initial decrease in pixel brightness followed by a period of brightness increase. Panels **(b,c)** are measurements on current extension growth only and both show refilling following air injectionasthe initial decrease in pixel brightness is followed by an increase in pixel brightness. Dashed lines reflect the initial (prior to embolism induction) and the maximum final (recovered) levels of pixel brightness.

The temporal and spatial dynamics of embolism were measured using MRI on intact, well hydrated *A. rubrum* plants that were exposed to air-pressurization treatments that created a 5.0-MPa pressure gradient across the xylem bordered pit membranes. Changes in the average pixel brightness of analyzed tissues were used to assess changes in stem water content in two regions of the stems during these pressurization treatments: (1) current-year extension growth (one xylem ring) and (2) 1-year-old stems (two xylem rings). As expected, air injection treatments, that created a 5.0-MPa gas/water interface pressure differential at the bordered pit level, resulted in the loss of pixel brightness and was interpreted as a drop in the water content in the stem and formation of embolism. In 1-year-old stems, embolism formed in both the older (internal ring) and in the current-year xylem (outer ring). The loss of water content determined from decreased pixel brightness in the older ring was found to be much more pronounced (**Figure 2**). We did not observe any signs of brightness recovery over a 10-h measurement period in the older ring. However, the initial loss of water content in current-year xylem recovered within 2 h from induction of embolism (**Figure 2**). In two other instances, only sections of the current extension growth of the stem were observed with the MRI, and we found that the initial drop of water content due to air-injection induced embolism was followed by recovery within a 1- to 2-h period (**Figure 2**). The spike in brightness during the air injection process reflects the movement of water in the xylem caused by the water being replaced with the air that is being forced into the xylem (**Figure 2**).

**FIGURE 3:**
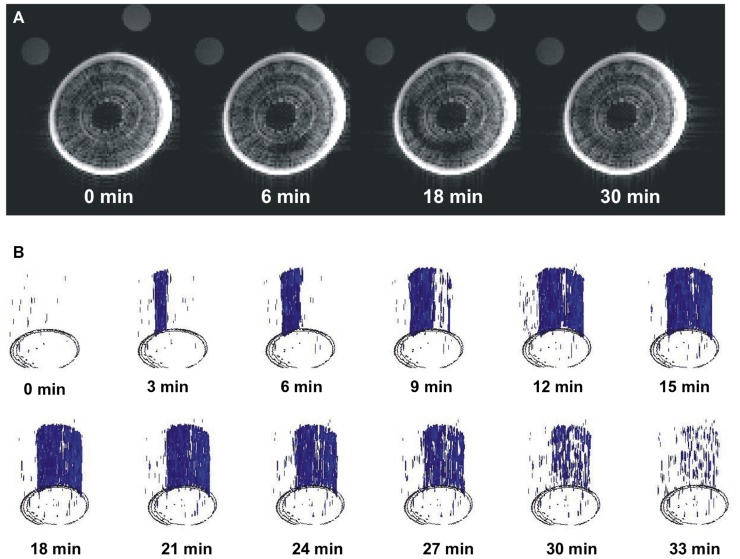
** Images of real-time MRI observations of a section of stem from an intact A. rubrum plant exposed to moderate levels of water stress. (A)** Images show the development of a spontaneously occurring embolism (at 6 min) and its spread across the current growth ring (at 18 min) followed by an almost full water content recovery (at 30 min). **(B)** Three dimensional analyses of embolism formation, its spatial spread, and recovery. The blue color denotes volumes with 90% loss of pixel brightness. Please note that to improve embolus visibility, images were reoriented in respect to the MRI images. Please consult the Supplementary Material online (Video S1 and S2).

The long-term MRI monitoring experiment was conducted on intact potted plants that were undisturbed for 10–15 h each. This experiment was designed to determine if *A. rubrum* plants undergo “natural” spontaneous embolism formation within their xylem on plants exposed to moderate levels of water stress (xylem water potentials of about -0.5 MPa). In four out of the five plants, we observed no dramatic changes in pixel brightness. We observed small levels of brightness flickering in some pixels that appeared across the xylem tissue. These brightness changes were considered to be potential artifacts or possibly changes in water content of the xylem fibers. Apart from these small flickering, we observedone large a spontaneous occurrence of a drop in pixel brightness in one plant. This event had a similar change in pixel brightness as observed in stems that were injected with air, suggesting that it was a large embolism event. The event started in the current-year xylem and it spread along the perimeter of the last year xylem annual growth ring. At its maximum, the embolized areacovered 1/3 of the stem perimeter. The change in pixel brightness along the stem length (15 mm) occurred simultaneously, i.e., faster than the 180 s time interval between consecutive images. The radial spread of the embolism was slower and took several minutes to move along the stem perimeter. The drop in pixel brightens (or embolism event) was followed by a rapid increase in brightness, implying that unbound “free water” was moving back to the embolized section. It took 20 min for the pixel brightness to return to near initial level. It should also be noted that the reappearance of water was not instantaneous along the stems length and that the stem cross sections being monitored with the MRI returned to their initial pixel brightness at different times (**Figure 3**; Video S1 in Supplementary Material). There was no noticeable directionality in the observed refilling process, in that it seemed to be occurring randomly across the stem segment (**Figure 3B**; Video S2 in Supplementary Material).

## DISCUSSION

Magnetic resonance imaging provides a means of viewing the xylem sap directly within intact plants. However, only a few studies have used this method to investigate plant embolism/refilling cycle because of technical limitations related to its spatial and temporal resolution (Köckenberger et al., 1997; Holbrook et al., 2001; Clearwater and clark, 2003; Utsuzawa et al., 2005; Scheenen et al., 2007; Kaufmann et al., 2009; Van As et al., 2009). The limits on spatial resolution (>50 mm) resulted in all previous studies being focused on vine species with large vessels (Holbrook et al., 2001; Clearwater and clark, 2003; Kaufmann et al., 2009). More over this vessel level resolution could only be achieved with long acquisition times thus limiting temporal resolution to tens of minutes between consecutive images. Use of very high magnetic strength magnet (e.g., >11 T) can help to overcome some of these limits but the trade-off between resolution and frequency of image collection would remain a valid problem for observations of embolism at the level of single vessel in trees characterized by vessels diameter less than 50 μm. Here we have shown that in diffused porous species with small vessels one can use average pixel brightness of xylem as a measure of water content in stem and that pattern of brightness change in response to water stress is well correlated with pattern of percent loss of stem conductivity (PLC; **Figure 1**). Thus we suggest that low resolution MRI analysis can be successfully used to determine dynamic changes of stem hydraulic properties even when one cannot resolve single vessels. This opens venue to *in vivo* analysis of hydraulic dynamics of trees with small vessels that were shown to undergo embolism-refilling cycles (Salleo et al., 1996).

We applied this low spatial – high temporal resolution approach to make observations on stem samples that were subjected to air-pressurization treatments – induced embolism (**Figure 2**). The application of pressurized air into the stem resulted in water loss from the xylem in both the current and in the older growth ring (as seen by loss of average pixel brightness). Following depressurization, we observed refilling (within 1 h), but only in the current-year xylem. The older, inner wood ring remained embolized despite monitoring the stem in the MRI for more than 12 h post air-injection treatment. These data provides insight into the functional differences between current (new) and older wood. The current-year xylem in maple has been shown to be the least vulnerable part of the xylem to embolism formation (Melcher et al., 2003; Choat et al., 2005), and the MRI data presented here, suggest that it is also protected from failure by the ability to refill. It is possible that the older wood refills when the entire plant is relieved from water stress conditions (following rain event). If this is true, then the plant may use the older xylem as a water capacitor (Meinzer et al., 2003). Since the older xylem is more susceptible to embolism formation, it would provide plants with a mechanism to release water from the old xylem to the current-year xylem during times of water stress.

In this report, we also describe continuous observations of the water status of the xylem of *A. rubrum* stems exposed to low levels of water stress (~-0.5 MPa). During the 60 h of MRI observations, we were only able to observe one embolism formation event. However, we feel confident that this one observation placed in the context of other available data (see Brodersen and McElrone, 2013) provides strong support for *in situ* formation of embolism in stems of moderately stressed plants. In addition, our data provide evidence of rapid xylem refilling as the loss of pixel brightness (formation of embolism) was followed by the restoration of pixel brightness (refilling) to pre-embolism conditions in the affected area with 30 min. The spread of this naturally occurring embolism event was limited only to current-year xylem. The circumferential progress (several millimeters) occurred over several minutes (i.e., over several consecutive images) while vertical occurrence (3 cm distance) was instantaneous (i.e., within time needed to collect signal for a single image). Analysis of refilling showed no directionality and water seemed to occur in many separated image voxels across entire embolized volume.

The unique *in vivo* time-lapse observation of embolism/refilling cycles using MRI highlights important considerations for our current understanding of xylem function, i.e., that cavitation might be an everyday event in the stems of transpiring plants at a frequency that is related to tension of sap in the xylem. As our data show, the restoration of water content in the affected stem occurred relatively quickly, but one can expect that the effectiveness of refilling would decrease with increasing levels of water stress. This would eventually lead to a situation when embolisms may not be removed by refilling, and that embolisms may accumulate faster than they can be refilled, resulting in an increase of non-functional conduits (Secchi and Zwieniecki, 2012). Thus, we can expect that the current level of embolisms in a stem is a product of the probability of embolism occurrence (positively related to tension) and embolism-refilling rate which is inversely related to tension and the ability of the plant to supply energy (Zwieniecki and Holbrook, 2009; Nardini et al., 2011; Secchi and Zwieniecki, 2012). If this view is correct, then it would be predicted that plants continuously go through embolism/refilling cycles in different parts of the stem and that the percent of measured embolisms reflects the current balance between these two processes.

It is interesting to note that the rate of refilling in *A. rubrum*, from both spontaneous cavitation, and from the air-injection method was fast (less than an hour). This stands in contrast to the observation of refilling of embolized vessels in *Vitis *spp.(Brodersen et al., 2010) where refilling extended for several hours and in *C. sativus *which took 17–47 h (Scheenen et al., 2007). The discrepancy may be the result of the vessel volume differences between the studied species. Maple vessels (this study) are general less than 50 μm in diameter, while the grapevine vessels are often more than 200 μm in diameter (Brodersen et al., 2010). This difference results in roughly 16 times larger volume of the grapevine vessels compared to maplevessels per length. Thus, if the living cell refilling activity is not enhanced, then we might expect that the time required to refill grapevine vessels will be 16 times longer (i.e., ~8 h), which is very similar to the time reported by Brodersen et al. (2010). The same might be true for the time discrepancy from this and the Scheenen et al. (2007) study as they were focusing only on the largest vessels in the cucumber stem (~200 μm in diameter). It is also possible that difference in temporal dynamics of refilling reflect intrinsic physiological differences between herbaceous annual plants and woody perennials, where perennials may need mechanim for fast refilling to ensure long-term xylem functionality.

In conclusion, comparative analysis of the hydraulic determination of PLC and pixel brightness from MRI images in relation to stem water potential showed a functional paralelism allowing for interpretation of MRI data in the context of PLC without need resolve single vessels. Further, this work provides visual evidence that the embolism formation/refilling cycle exists in intact *A. rubrum *stems experiencing moderate levels of water stress. The long-term observations using MRI of undisturbed stems of *A. rubrum* showed one such unambiguous event that in combination with other reports (Brodersen and McElrone, 2013) provide support for the existence of rapid refilling in moderately stressed plants. However, the functionality (restoration of water transport) of vessels refilled under tension still remains unanswered.

## Conflict of Interest Statement

The authors declare that the research was conducted in the absence of any commercial or financial relationships that could be construed as a potential conflict of interest.

## Supplementary Material

The Supplementary Material for this article can be found online at: http://www.frontiersin.org/Plant_Biophysics_and_Modeling/10.3389/fpls.2013.00265/abstract

Video 1 **A time lapse video of *A. rubrum* stem from an MRI observation**. The video shows a natural occurrence of embolism in an intact stem of a potted tree. The embolism occurs in the current-year vascular ring, spreads and then disappears as refilling takes place.

**A 3D reconstruction of embolism dynamics in a stem of *A. rubrum* from images collected during MRI observations**. Five vertical observation planes (1.5 mm thick) separated by 1.5 mm distances are shown.
